# Harnessing health plan enrollee data to boost membership in patient-powered research networks

**DOI:** 10.1186/s12913-020-05325-z

**Published:** 2020-05-25

**Authors:** Xiaoxue Chen, Abiy Agiro, W. Benjamin Nowell, Sara Loud, Robert McBurney, Kalen Young, Rebecca Sutphen, Elizabeth Bourquardez Clark, Cristina M. Burroughs, Jeffrey R. Curtis, Antoine G. Sreih, Peter A. Merkel, Kevin Haynes

**Affiliations:** 1 HealthCore, Inc., 123 Justison Street, Suite 200, Wilmington, DE 19801-5134 USA; 2 Global Healthy Living Foundation, Upper Nyack, New York, NY USA; 3 Accelerated Cure Project, Waltham, MA USA; 4 Vasculitis Foundation, Kansas City, MO USA; 5 University of South Florida, Tampa, FL USA; 6 Division of Clinical Immunology and Rheumatology, University of Alabama at Birmingham, Birmingham, AL USA; 7 Division of Rheumatology and Department of Biostatistics, Epidemiology, and Informatics, University of Pennsylvania, Philadelphia, PA USA

**Keywords:** Patient-powered research networks, Anonymous linkage methods, Outreach, Patient participation

## Abstract

**Background:**

Patient-powered research networks (PPRNs) have been employing and exploring different methods to engage patients in research activities specific to their conditions. One way to intensify patient engagement is to partner with payer stakeholders. The objective of this study was to evaluate the effectiveness of two common payer-initiated outreach methods (postal mail versus email) for inviting prospective candidates to participate in their initiatives.

**Methods:**

This descriptive study linked members of a nationally-representative private insurance network to four disease-specific PPRN registries. Eligible members meeting diagnostic criteria who were not registered in any of the four PPRNs by 02/28/2018 were identified, and randomly assigned to either the mail or email group. They were contacted in two outreach efforts: first on 04/23/2018, and one follow-up on 05/23/2018. New registration rates by outreach method as of 8/31/2018 were determined by relinking. We compared registrants and non-registrants using bivariate analysis.

**Results:**

A total of 14,571 patients were assigned to the mail group, and 14,574 to the email group. Invitations were successfully delivered to 13,834 (94.9%) mail group and 10,205 (70.0%) email group members. A small but significantly larger proportion of mail group members, (*n* = 78; 0.54, 95% Confidence Interval [CI] {0.42–0.67%}) registered in PPRNs relative to the email group (*n* = 24; 0.16, 95% CI {0.11–0.25%}), *p* < 0.001. Members who registered had more comorbidities, were more likely to be female, and had marginally greater medical utilization, especially emergency room visits, relative to non-registrants (52.0% vs. 42.5%, *p* = 0.05).

**Conclusion:**

A health plan outreach to invite members to participate in PPRNs was modestly effective. Regular mail outperformed less costly email. Providing more value-add to participants may be a possible way to increase recruitment success.

## Background

Since Patient-Centered Outcomes Research Institute (PCORI) established the Patient-Centered Research Network (PCORnet) [1–3], the 20 Patient-Powered Research Networks (PPRNs), 13 Clinical Data Research Networks (CDRNs), and 2 Health Plan Research Networks (HPRNs) [1–3] have actively engaged in harnessing data from multiple sources for more effective, efficient, and patient-centered research [1–3]. PPRNs, patient-led organizations focused on a particular heath condition, are organized around the evidence needs of patients. The 2013 funding mandate of PPRNs empowered PPRNs to create and sustain scalable data systems using proficient and reliable technological standards that are sufficiently reliable to share with other PCORnet affiliates, and are capable of generating high-quality information suitable for use in research [4].

Outreach activities are the life force for the creation and nurturing of a readily available pool of people motivated to participate in research opportunities relevant to their health condition(s) [4]. Several approaches by PPRNs include cooperation with communities, faith-based groups, social clubs and service organizations, hospitals, physician offices, and pharmacies feature commonly in the outreach and engagement strategies. PPRNs also make language and literacy appropriate recruitment and educational materials in various forms (including print, electronic, audio, and video streaming services) to reach historically under-represented patient groups in terms of race/ethnicity, socioeconomic status, geographic location, health literacy, and clinical severity [2].

Furthermore, as part of a range of activities to broaden their membership and enlarge their research pursuits in disease categories they focus on, PPRNs have sought to intensify their engagement with payer stakeholders, which specifically included PCORnet’s HPRNs, based on shared research goals and interest in patient outcomes. Despite challenges endemic to recruitment and retention processes, such collaborations between PPRNs and HPRNs embody essential characteristics for success. The biggest strength is the capability to easily and accurately identify a large patient candidate pool from health plans’ data under minimal efforts.

In a prior study [5], we described linkage between 4 PPRNs and a network of 14 nationally-representative health plans. This previous work demonstrated successful data linkage of payer-based administrative claims data with PPRN registry data and that claim-based disease phenotypes can accurately identify patients with conditions of interest (when compared against patient self-reported conditions in PPRN registry data). Building upon our previous work, we hypothesized that a health plan could engage their membership with information about the PPRNs and enhance current patient recruitment activities conducted by PPRNs [6]. The objective of this study was to evaluate two common payer-initiated outreach methods for inviting prospective candidates to participate in their initiatives: regular postal service mail (mail group) and Internet-based electronic mail (email group). Our main goal was to evaluate the primary outcome of this outreach effort: the number of health plan members who newly registered to participate in any of the four PPRNs of interest.

## Methods

### Study design and data sources

In this descriptive study, health plan enrollee data were queried from the HealthCore Integrated Research Environment (HIRE®) to identify members who met computable phenotypes (a set of claim-based algorithms) between Jan 1, 2006 and February 28, 2018 but who were not already registered in any of the four disease-specific PPRNs of interest: ArthritisPower (rheumatoid arthritis, psoriatic arthritis and psoriasis, and spondyloarthritis), ABOUT (American BRCA Outcomes and Utilization of Testing), iConquerMS (Multiple Sclerosis Patient-Powered Research Network) or the VPPRN (Vasculitis Patient-Powered Research Network). The HIRE is a repository of consistently updated longitudinal patient-level administrative claims data for approximately 60 million commercially insured health plan enrollees, with 13 million members currently enrolled at the time of the study outreach, from 14 major regional health plans across the continental United States [7]. Members’ PPRN registration status was obtained using privacy-preserving record linkage of overlapping health plan and PPRN members with secure HIPAA-compliant, one-way, cryptographic hash functions outlined in a prior publication [5]. Members’ most recent email and mail address was obtained from plan data and used for outreach.

The computable phenotypes or coding algorithms were obtained directly from the PPRNs. These algorithms were developed by the PPRNs with patient and clinician engagement in the formation of the PPRNs, and further validated in a prior study [5]. In the ArthritisPower PPRN, the computable phenotype was based on at least two medical outpatient specialist claims, such as a dermatologist for psoriasis or a rheumatologist for other relevant arthritis conditions [8]. In the iConquerMS PPRN, the computable phenotype relied on at least three claims for Multiple Sclerosis (MS)-diagnosis related hospitalizations or MS-diagnosis related outpatient/Emergency Department (ED) visits or MS-related prescription fills in any combinations that were less than 12 months apart [9]. The computable phenotype in the ABOUT PPRN consisted of at least two diagnoses of breast or ovarian cancer in medical claims that were 30 days apart in the clinician office setting [10]. The computable phenotype in the Vasculitis PPRN consisted of a combination of diagnosis codes, physician specialty (rheumatology, immunology, nephrology, otorhinolaryngology or pulmonary, cardiology or vascular surgery), and the use of immunosuppressive medications [11].

This prospective randomized study received New England Institutional Review Board (IRB) approval, and subsequent enrollment into respective PPRNs was governed by individual IRBs governing the respective PPRNs. Researchers only accessed a limited data set of member information strictly relevant to the purposes of this study. All research materials and processes complied with the applicable privacy policies specified in the Health Insurance Portability and Accountability Act (HIPAA).

### PPRNs of interest

Seven PPRNs were initially selected to engage to ensure that we had a diverse representative sample of PPRNs; four PPRNs agreed to participate in the study. The four PPRNs in this study are managed by combined investigator and patient governance groups, and are a part of the PCORnet.

- The ABOUT Network focuses on hereditary breast or ovarian cancer (aboutnetwork.org). Its membership of more than 6500 participants includes both males and females older than 18 years of age, and participants may have a known genetic mutation (within their family) or a personal or family history of breast, ovarian, or related cancers [12].
- The ArthritisPower PPRN concentrates on musculoskeletal and inflammatory skin conditions (particularly arthritis or psoriasis), and manages a nationwide research registry network of more than 17,000 patients diagnosed with rheumatoid arthritis, psoriatic arthritis and spondyloarthritis (e.g., psoriatic arthritis and ankylosing spondylitis) along with a variety of other rheumatic conditions [13].
- The iConquerMS PPRN, which specializes in multiple sclerosis (MS), is a community of more than 4000 participants. People with MS and other stakeholders enroll on the network’s portal, iConquerMS.org, which facilitates the collection of demographic information, MS history and patient-reported outcomes data, and maintains ongoing interactions and communications with the network’s members [14].
- The VPPRN has more than 2500 members who are enrolled in clinical studies investigating multiple types of vasculitis. The VPPRN is a partnership of the Vasculitis Foundation and the Vasculitis Clinical Research Consortium [15].

### Engagement plan

The setting for this study included the HIRE data repository for patient identification and the web portals of the four PPRNs for recording electronic consent. The population eligible for engagement consisted of currently active members in the 14 health plan networks, curated in the HIRE database, who were identified using the validated computable phenotype of interest and who were not already current members of one of the four PPRNs. All eligible members with both home and email addresses were randomly (1:1) assigned to the mail or the email group stratified by condition of interest for each PPRN. An experienced, external vendor with an established business relationship with HealthCore was contracted to plan the logistics and conduct the outreach activities to the identified health plan members via the assigned modality. The first outreach to members was on April 23, 2018, and the follow-up communication was sent 1 month later, on May 23, 2018. The mail included health plan logo on the envelope and the signatory of the letter by the health plan medical director. Subject line of the email was “Health Research Opportunity- Your Chance to Make a Difference!”. Both mail and email had the opening sentence “Name of the member, you can influence the future of health care”. Representative content of both the initial and reminder mail or email drop is shown in Supplemental materials.

### Outcomes

The primary outcome was the number of new registrants out of the health plan members identified and outreached, defined as obtaining an electronically signed consent form for patient participation in one of the four participating research PPRNs by August 31, 2018.

We determined the primary outcome by conducting privacy-preserving record linkage across the four PPRNs after August 31, 2018 to determine which members joined a respective PPRN.

### Statistical analysis

Intent-to-treat approach was used to analyze our data; hence, we had included patients whose mail or email communication could not be delivered (emails bounced back) and patients who appeared in the Do-not-contact database (post randomization but prior to the intervention). In a sensitivity analysis, we also used the as-treated approach, by which we mean a population of members who mail and email communications were not returned and who didn’t show up in the Do-not-contact database prior to our intervention. We used descriptive statistics to establish patient counts and to evaluate demographic and clinical characteristics including age, sex, region, comorbidities (measured by the Deyo Charlson comorbidity index) [16], and medical and pharmacy utilization during all available health insurance coverage period, January 2006 through February 2018. Registration rates were calculated as simple proportions, and the exact 95% confidence intervals (CI) for all registration rates were determined with binomial random variables. A conventional alpha of 0.05 was used to interpret statistical significance. Statistical analyses were performed with SAS EG 7.1 (SAS Institute, Cary, NC).

## Results

### Study population

**Fig. 1:**
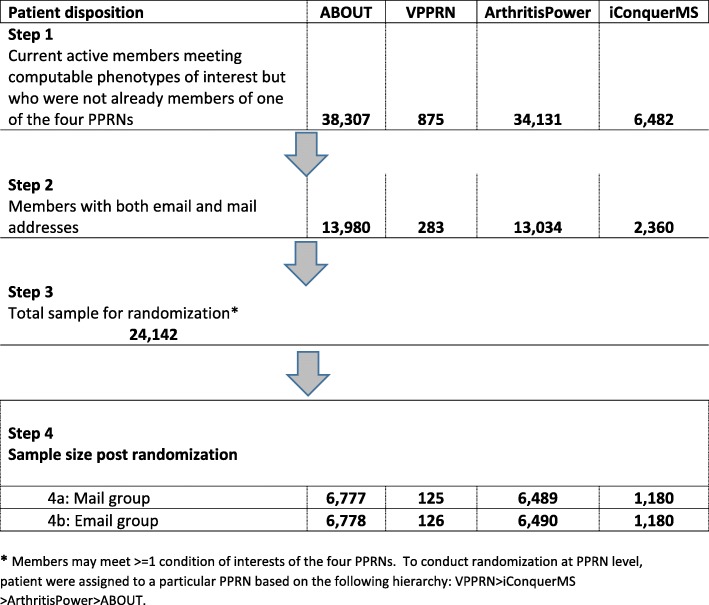
Patient inclusion/exclusion and final study population

Of the 29,142 members who met one of the computable phenotype definitions, half (*n* = 14,571) health plan members were randomly assigned to the mail group and the remainder (*n* = 14,574) to the email group. The randomization stratified by computable phenotype of interest for each PPRN generated a difference of three people in the overall denominator count of mail and email groups (Fig. 1).

Overall, 728 (5.0%) of the randomized patients targeted to receive regular mail and 709 (4.9%) for email had existing profiles of Do Not Contact (DNC) requests, as shown in Table 1. Undeliverable addresses (returned emails or mails) were considerably fewer in the mail group, 9 (0.1%), relative to email group, 3546 (24.3%). As a result, invitations were delivered to 13,834 (94.9%) members in the mail group and 10,319 (70.8%) in the email group.

**Table 1:** Study sample post randomization

	Mail group		Email group	
	N	%	N	%
Total sample for outreach after randomization	14,571	100%	14,574	100%
Members with undeliverable home or email address	9	0.1%	3546	24.3%
Members in Do-not-contact database prior to intervention	728	5.0%	709	4.9%
Members with deliverable home or email address	13,834	94.9%	10,319	70.8%

### Health plan member demographics and clinical characteristics

Table 2 outlines the demographic and clinical characteristics of the study population. Mean age (SD) was similar for the mail group, 50.8 (10.5) years and email group, 50.7 (10.5) years. In excess of two-thirds of the members were in the 45–64 year age group, with 10,073 (69.1%) in the mail group and 10,010 (68.7%) in the email group. Females constituted 78.4 and 78.5% of the mail and email groups, respectively. Residential location was predominantly urban, with 80.8% in the mail group and 81.3% in the email group, with smaller proportions 18.3 and 17.9% in rural areas, respectively. Slightly larger proportions of members were located in the Midwest (37.6 and 38.4%) than the West (30.4 and 30.0%) and the South (24.0 and 24.0%) in the mail and email groups, respectively. The two groups had similar health status as reflected by Deyo-Charlson Comorbidity Index total and severity scores, and comparable medical utilization history.

**Table 2:** Characteristics of health plan members invited to join a Patient-powered research network

Health plan member characteristics	Mail group		Email group		***P*** values
	Mean/N	SD/%	Mean/N	SD/%	
N	14,571		14,574		
Age, mean (SD)	50. 8	10.5	50.7	10.54	0.50
**Age category (years),** n (%)					0.55
18–20	41	0.3	46	0.3	
21–44	3707	25.4	3797	26.1	
45–64	10,073	69.1	10,010	68.7	
65 and over	750	5.1	721	4.9	
**Female,** n (%)	11,424	78.4	11,440	78.5	0.85
**Residential location** (based on zip code)**,** n (%)					0.27
Urban	11,772	80.8	11,845	81.3	
Rural	2663	18.3	2615	17.9	
Unknown	136	0.9	114	0.8	
**Health plan coverage,** n (%)					0.14
Commercial	14,182	97.3	14,224	97.6	
Medicare Advantage	389	2.7	350	2.4	
**Census Region,** n (%)					0.37
Northeast	1164	8.0	1110	7.6	
Midwest	5475	37.6	5599	38.4	
South	3498	24.0	3497	24.0	
West	4434	30.4	4368	30.0	
**Clinical History**^**a**^					
Comorbidity score (Deyo-Charlson Index), mean (SD)	2.17	2.62	2.15	2.61	0.29
Comorbidity score category**,** n (%)					0.46
0	4347	29.8	4406	30.2	
1 or 2	6100	41.9	6136	42.1	
3 or more	4124	28.3	4032	27.7	
**Medical Utilization History (all cause)**^**a**^					
Any hospitalization, n (%)	4432	30.4	4459	30.6	0.74
Any emergency room visit, n (%)	6150	42.2	6244	42.8	0.27
Any Outpatient visit, n (%)	14,571	100.0	14,573	100.0	0.32
Primary care physician visits, n (%)	12,984	89.1	12,955	88.9	0.55
Specialist visits, n (%)	14,458	99.2	14,476	99.3	0.30

*SD* Standard deviation

^a^characteristics measured using all available coverage period from January 2006 to February 2018

### Health plan member registration by randomization group

As shown in Table 3, a small but significantly larger proportion of members from the mail group (*n* = 78 out of 14,571; 0.54, 95% Confidence Interval [CI]; {0.42–0.67%}) registered in one of the PPRNs relative to the email group (*n* = 24 out of 14,574; 0.16, 95% CI {0.11–0.25%}), *p* < 0.001. In the sensitivity analysis excluding members with undelivered email/mail and those with existing profiles of DNC request, the mail group(*n* = 78; 0.56, 95% CI;{0.45–0.70%}) remained more likely to register in PPRNs relative to the email group (*n* = 23; 0.22, 95% CI {0.14–0.33%}), p < 0.001.

**Table 3:** Rate of health plan member registration^a^ by randomization group^b^

	Mail group (***N*** = 14,571)			Email group (***N*** = 14,574)			P values
Intent-to-treat approach	N	%	95% CI	N	%	95% CI	
Patient engaged by the intervention	78	0.54%	0.42–0.67%	24	0.16%	0.11–0.25%	<.001
As treated approach^c^	*N* = 13,834			*N* = 10,319			
Patient engaged by the intervention	78	0.56%	0.45–0.70%	23	0.22%	0.14–0.33%	<.001

^a^Plan member registration = Invited health plan member who joined a PPRN

^b^Randomization group = Invited health plan members were randomized to either mail or email outreach

^c^Patients with undeliverable home or email address and those in existing Do-Not-Contact list were excluded in the as-treated analysis

The heterogeneity across PPRNs is presented in Supplemental table 1. The mail group had statistically significant higher registration rate than email group in ABOUT and ArthritisPower. The numbers were too small to conclude in VPPRN and MS-PPRN.

### Characteristics of members who registered in PPRNs vs. non-registrants

The proportion of females who registered was greater than female non-registrants, 87.3% versus 78.4%, respectively, *p* = 0.03. Health plan members with more comorbidities were somewhat more likely to register in PPRNS as indicated by greater Deyo-Charlson Comorbidity Index scores for patients who registered compared to those who did not, 2.5 (2.79) versus 2.2 (2.61), *p* = 0.08. Members who registered with PPRNs had marginally higher medical utilization compared to non-registrants including emergency room visits, 52.0% vs. 42.5%, *p* = 0.05, as shown in Table 4.

**Table 4:** Characteristics of health plan members who did or did not register in a Patient-powered research network

Health plan member characteristics	Registered		Not registered		***P*** values
	Mean/N	SD/%	Mean/N	SD/%	
N	102		29,043		
Age, mean (SD)	51.8	10.09	50.7	10.52	0.17
**Age category (years)**, n (%)					0.41
18–20	0	0.0	87	0.3	
21–44	20	19.6	7484	25.8	
45–64	78	76.5	20,005	68.9	
65 and over	4	3.9	1467	5.1	
Female, n (%)	89	87.3	22,775	78.4	0.03
**Residential location** (based on zip code), n (%)					0.17
Urban	77	75.5	23,540	81.1	
Rural	25	24.5	5253	18.1	
Unknown	0	0.0	250	0.9	
**Health plan coverage**, n (%)					0.37
Commercial	98	96.1	28,308	97.5	
Medicare Advantage	4	3.9	735	2.5	
**Census Region**, n (%)					0.23
Northeast	9	8.8	2265	7.8	
Midwest	48	47.1	11,026	38.0	
South	19	18.6	6976	24.0	
West	26	25.5	8776	30.2	
**Clinical History**					
Comorbidity score (Deyo-Charlson Index), mean (SD)	2.5	2.79	2.2	2.61	0.08
Comorbidity score category, n (%)					0.01
0	17	16.7	8736	30.1	
1 or 2	53	52.0	12,183	41.9	
3 or more	32	31.4	8124	28.0	
**Medical Utilization History (All cause)**					
Any hospitalization, n (%)	34	33.3	8857	30.5	0.53
Any emergency room visit, n (%)	53	52.0	12,341	42.5	0.05
Any Outpatient visit, n (%)	102	100.0	29,042	100.0	0.95
Primary care physician visits, n (%)	94	92.2	25,845	89.0	0.31
Specialist visits, n (%)	101	99.0	28,833	99.3	0.76

Mail and email registrants had similar baseline characteristics as shown in Supplemental table 2.

## Discussion

Recruitment of patients for research studies can be challenging and require expenditure of time and resources [17]. Our study was among the first studies to examine the effectiveness of payer-initiated recruitment efforts to engage patients with PPRNs [18]. Using computable phenotypes that can accurately identify patients with conditions of interests from health plan’s data, this study examined the registration rates of patients who were invited by health plans through email or email to register and participate in patient-focused research organizations that focus on health conditions directly applicable to them; such participation could potentially lead to enrollment in clinical studies. The yield of this approach, the number of new registrants to one of the four research PPRNs, were modest.

The main outcome in this study, patient registration in PPRNs after a 3-month outreach period, was substantially lower than 1% for both the mail group (0.54%) and the email group (0.16%). While no prior data for the results of PPRN outreach via mail versus email is available for a direct comparison, results from other outreach activities appear consistent with our finding: direct mail outperforms email outreach. In a 2015 report on survey response rates from 485 industry respondents, Direct Marketing Association (DMA) reported a response rate of 3.5% for direct mail which is seven-fold greater than the response in our study [19]. For emails, however, DMA reported only 0.1% response, which is less than responses in our study. Despite the differences in study samples and study purposes (survey response vs actual registration), postal service mail outperformed email via the internet in both cases. These results suggest that while emails are less costly they seem less effective in eliciting responses than direct mail. These techniques can be more effective when used in combination, especially if supported by telephone outreach, a combination for which a systematic review reported an absolute 6% improvement in recruitment of patients to trials [20, 21].

Interestingly, a partnership between ABOUT PPRN and Aetna mailed recruitment information to commercial health plan members whose clinicians had ordered BRCA testing [22]. They invited patients to complete a questionnaire and further requested their consent to recontact them for further research activities. The study included $5 bill as a prepaid token of appreciation and reported 34.7% survey response rate. Unlike responding to a survey, our study was looking at the recruitment of members through a health plan outreach inviting them to join a patient-powered-research network. Alexander et al.’s mailing intervention study which invited patients to participate in an online health program might be a better comparison to our study [23]. Our enrollment rate was more in line with the rate reported in this online intervention program (4.3%). The online intervention program also reported the enrollment rate ranging from 1 to 11% by incentive combination, suggesting incentive was a big influencer in patient decisions in participation. Nevertheless, the partnership between ABOUT PPRN and Aetna and our study present the unique role of payers in engaging patients with patient-centered research activities [20].

Patient registration rate in the email group was 0.16% in the main (intent-to treat) analysis as opposed to 0.22% in the as-treated analysis. One contributor to lower email results is the greater likelihood that emails are fraught with more undeliverable addresses than a direct mail. This may be a reflection of the ease of abandoning or changing an email address based on evolving preferences for Internet service providers or email hosting services versus changing a mailing address, which typically necessitates a physical move. Randomization ensured balance on existing profiles of DNC requests between the email and the mail group prior to plan outreach. In the post-outreach period, 114 new DNC requests were documented for the email group versus none for the mail group. Although DNC requests may be more a reflection of personal virtues and preferences on individuals versus anything inherent in the outreach method, the exclusive DNC requests in the email group suggested that the ease of communications via email played a big role.

As might be expected, health plan members who registered appeared to have more comorbidities in addition to the main targeted disease(s) of interest, and the greater use of medical services, especially emergency room visits. There are continuing debates around gender’s difference in participation in trials [24, 25]. In our study with a large representation of women due to the nature of the conditions of interest, women were more likely to register and participate in PPRNs than men.

As reflected by the relatively modest registration rates in this study, and for outreach efforts in general, one of the urgent quests is to find ways to improve participation rates. This topic has been studied broadly in survey methodologies. Monetary and non-monetary incentives, personalized letters, mixed-modes of follow-up, assurance of confidentiality all have shown to improve the response rate of survey outreach and are potentially applicable to outreach efforts more broadly [23, 26, 27]. Unfortunately, budget and legal concerns have prevented from us employing most of these more effective strategies. Singer et al. dived deeper into the role of perceived benefits and costs in patients’ medical decisions and concluded that benefit-cost calculations did inform patients’ medical decision making [28]. Similarly, cost-benefit considerations have been shown to influence decisions to participate in research activities or interventions [29–31]. Therefore, determining the most effective methods for member engagement requires solid understanding of risk factors affecting patients’ health, and the non-healthcare factors that affect how they seek care and respond to health plans and providers. For this reason, joining a research study may lack appeal if there is little perceived value for the health of the participant, because altruism becomes pitted against hassle, concerns for security and privacy, etc. Providing more value-add to participants may be possible and could increase recruitment success. For example, the results of the study might be returned back to the patient and his/her medical provider, with the possibility that the information could be used to inform and even improve the patient’s own healthcare. In this example and many others, the lines between research and high quality, high-touch integrated care could be blurred, so that patients can maximize the benefit of their participation.

### Limitations

The study only examined patient registration and participation in PPRNs after email/mail outreach; none of the intermediate steps (e.g. whether the patient opened the email/mail or went to the registration webpage) was captured. Such information is critical to optimize future recruitment efforts. Although computable phenotypes can accurately identify patients with conditions of interest, inherent features of secondary data, administrative claims in this study, can be affected by coding inaccuracies and result in outcome misclassifications, and inaccurate estimation of patient populations. Thus, it’s possible that some patients received the invitations may not have the conditions of interest. This study employed deterministic matching, linkage methodologies, and hashing to identify overlapping members in PPRNs and health plans. While highly effective, this approach has notable limitations that were discussed by this team of authors in a companion publication [5]. For example, such deterministic matching techniques may miss patients who actually joined PPRNs where differences in spelling occur, resulting in possible underestimation of the study effect. The PPRNs may have been concurrently engaging in their own recruitment efforts within the same period. This may result in overestimation of the overall study impact but little influence on the comparison of email and mail outreach. Furthermore, the population in this study was commercially insured precluding ready generalizability of these results to different patient populations such as those insured by Medicare and Medicaid, who may have both greater or lesser motivations and different barriers to participate.

## Conclusions

The main thrust of this study, to invite health plan enrollees diagnosed with specific diseases of interest to register with disease-based PPRNs, was modestly successful. The study showed that outreach by regular postal service mailing marginally outperformed email outreach. Providing more value-add to participants may be a possible way to increase recruitment success.

## Supplementary information

**Additional file 1.**

## Data Availability

The datasets generated and analyzed during the current study are not publicly available due to containing information that could potentially identify study participants, but de-identified versions are available from the corresponding author on reasonable request.

## Abbreviations

PCORI: Patient-Centered Outcomes Research Institute
PCORnet: Patient-Centered Research Network
PPRNs: Patient-Powered Research Networks
CDRNs: Clinical Data Research Networks
HPRNs: Health Plan Research Networks
HIRE: HealthCore Integrated Research Environment
HIPAA: Health Insurance Portability and Accountability Act
ED: Emergency Department
MS: Multiple sclerosis
SD: Standard deviation
