# Supplementary material for: Harnessing health plan enrollee data to boost membership in patient-powered research networks
Source: BMC Health Serv Res. 2020 May 25;20:462. doi: 10.1186/s12913-020-05325-z (PMC7249317; doi:10.1186/s12913-020-05325-z)
Supplement: Supplementary file 1 — Additional file 1. [file 12913_2020_5325_MOESM1_ESM.docx]

**Supplemental Materials**

**Supplemental Table 1: Engagement heterogeneity across PPRNs**

|  | **Mail group** | | | **Email group** | | |  |
| --- | --- | --- | --- | --- | --- | --- | --- |
|  | n | N | % | n | N | % | P values |
| ABOUT | 29 | 6777 | 0.43% | 4 | 6778 | 0.06% | <0.001 |
| ArthritisPower | 39 | 6489 | 0.60% | 15 | 6490 | 0.23% | <0.001 |
| iConquerMS | 7 | 1180 | 0.59% | 4 | 1180 | 0.34% | 0.548 |
| VPPRN | 3 | 125 | 2.40% | 1 | 126 | 0.79% | 0.37 |

**Supplemental Table 2.Mail versus email health plan member characteristics**

| **Health plan member characteristics** | **Mail group** |  | **Email group** |  | **P values** |
| --- | --- | --- | --- | --- | --- |
|  | Mean/N | SD/% | Mean/N | SD/% |  |
| **N** | 78 |  | 24 |  |  |
| Age, mean (SD) | 52.5 | 9.93 | 49.7 | 10.54 | 0.24 |
| **Age category (years), n (%)** |  |  |  |  | 0.39 |
| 18 - 20 | 0 | 0.0 | 0 | 0.0 |  |
| 21 - 44 | 13 | 16.7 | 7 | 29.2 |  |
| 45 - 64 | 62 | 79.5 | 16 | 66.7 |  |
| 65 and over | 3 | 3.8 | 1 | 4.2 |  |
| **Female** | 67 | 85.9 | 22 | 91.7 | 0.46 |
| **Residential location (based on zip code), n (%)** |  |  |  |  | 0.54 |
| Urban | 60 | 76.9 | 17 | 70.8 |  |
| Rural | 18 | 23.1 | 7 | 29.2 |  |
| Unknown | 0 | 0.0 | 0 | 0.0 |  |
| **Health plan coverage, n (%)** |  |  |  |  | 0.94 |
| Commercial | 75 | 96.2 | 23 | 95.8 |  |
| Medicare Advantage | 3 | 3.8 | 1 | 4.2 |  |
| **Census Region, n (%)** |  |  |  |  | 0.93 |
| Northeast | 7 | 9.0 | 2 | 8.3 |  |
| Midwest | 38 | 48.7 | 10 | 41.7 |  |
| South | 14 | 17.9 | 5 | 20.8 |  |
| West | 19 | 24.4 | 7 | 29.2 |  |
| **Clinical History** |  |  |  |  |  |
| Comorbidity score (Deyo-Charlson Index) , mean (SD) | 2.54 | 3 | 2.25 | 1.98 | 0.78 |
| Comorbidity score category, n (%) |  |  |  |  | 0.45 |
| 0 | 15 | 19.2 | 2 | 8.3 |  |
| 1 or 2 | 39 | 50.0 | 14 | 58.3 |  |
| 3 or more | 24 | 30.8 | 8 | 33.3 |  |
| **Medical Utilization History (All cause)** |  |  |  |  |  |
| Any hospitalization, n (%) | 27 | 34.6 | 7 | 29.2 | 0.62 |
| Any emergency room visit, n (%) | 38 | 48.7 | 15 | 62.5 | 0.24 |
| Any Outpatient visit, n (%) | 78 | 100.0 | 24 | 100.0 | - |
| Primary care physician visits, n (%) | 71 | 91.0 | 23 | 95.8 | 0.44 |
| Specialist visits, n (%) | 77 | 98.7 | 24 | 100.0 | 0.58 |

**Supplemental material: Patient outreach letter**

[DATE] [INSERT HEALTH PLAN LOGO]

# [Subscriber First Name], you can influence the future of health care.

**This is your chance to share your opinions with top researchers and help steer the direction of treatment options for people with a personal or family history of hereditary cancers, vasculitis, multiple sclerosis or arthritis.**

**How to sign up**

**It’s easy to register for this research network opportunity. You’ll see a list of
four non-profit research groups on the back of this letter. Just reach out to the group that represents the health condition that interests you the most. That’s all there is to it.**

**What you need to know**

**The four groups listed on the back of this letter are working with PCORnet, a trusted research organization that focuses on helping people get the information they need so they can make the best possible decisions to improve their health.**

**You were selected to participate in this game-changing research network because you’re a valued [Full Brand Name] member, and not because of any health symptoms or conditions you might have.**

**[Subscriber First Name], your participation is extremely valuable. But if you decide you’d rather not take part in this research network, we’ll understand. And if you want to opt out of being invited to these research opportunities completely, just give us a call at [Opt Out Phone Number].**

**[Health Plan Medical Director Full Name]**

**Anthem Medical Director**

Here’s your chance to make a difference,
[Subscriber First Name].

**Your opinions will reach key decision-makers in health care, so please consider enrolling in a research network with any of the following groups.**

**Improving outcomes for people with or at risk for hereditary cancer**

**The ABOUT Network collects and shares real-world experiences of people with a family or personal history of breast, ovarian, prostate, melanoma, or pancreatic cancer, regardless of genetic-testing results.**

***
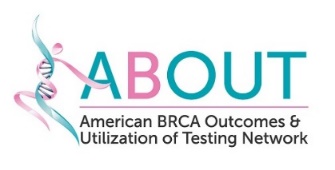
*Enroll at ABOUTnetwork.org, or email ABOUTNetwork@epu.usf.edu. You can also call (866) 632-2873.**

***
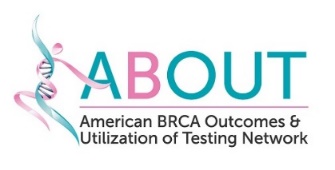
***

**Care and health for patients with vasculitis**


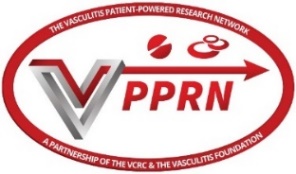
**The Vasculitis Network explores research questions that matter most to patients with vasculitis conditions. Visit vpprn.org to enroll and review a complete list of conditions. You can also email kyoung@vasculitisfoundation.org or call (866) 632-2873.**

**Multiple sclerosis (MS) research**


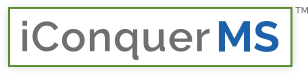
**iConquerMS is a people-powered online research initiative where people can securely contribute health information, insights, and ideas to advance MS research. Enroll at iConquerMS.org, or email info@iConquerMS.org.**

**Arthritis medication and non-medication treatment options**


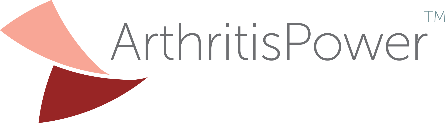
**ArthritisPower is a patient community that helps identify new treatment options through real-life experiences of people with arthritic conditions such as rheumatoid arthritis, ankylosing spondylitis, psoriatic arthropathy, or psoriasis. Enroll at ArthritisPower.org**

| **Patient disposition** | **ABOUT** | **VPPRN** | **ArthritisPower** | **iConquerMS** |
| --- | --- | --- | --- | --- |
| **Step 1**  Current active members meeting computable phenotypes of interest but who were not already members of one of the four PPRNs | **38,307** | **875** | **34,131** | **6,482** |
|  | | | | |
| **Step 2**  Members with both email and mail addresses | **13,980** | **283** | **13,034** | **2,360** |
|  | | | | |
| **Step 3**  Total sample for randomization*  **24,142** |  |  |  |  |
|  |  |  |  |  |
| **Step 4**  **Sample size post randomization** | | | | |
| 4a: Mail group | **6,777** | **125** | **6,489** | **1,180** |
| 4b: Email group | **6,778** | **126** | **6,490** | **1,180** |

***** Members may meet >=1 condition of interests of the four PPRNs. To conduct randomization at PPRN level, patient were assigned to a particular PPRN based on the following hierarchy: VPPRN>iConquerMS >ArthritisPower>ABOUT.
